# The Long-Term Effects of Pitavastatin on Blood Lipids and Platelet Activation Markers in Stroke Patients: Impact of the Homocysteine Level

**DOI:** 10.1371/journal.pone.0113766

**Published:** 2014-11-19

**Authors:** Hideki Sugimoto, Shingo Konno, Nobuatsu Nomoto, Hiroshi Nakazora, Mayumi Murata, Hisao Kitazono, Tomomi Imamura, Masashi Inoue, Miyuki Sasaki, Akihisa Fuse, Wataru Hagiwara, Mari Kobayashi, Toshiki Fujioka

**Affiliations:** Division of Neurology, Department of Internal Medicine, Toho University Ohashi, Tokyo, Japan; National University of Singapore, Singapore

## Abstract

To examine the impact of the plasma homocysteine level on the anti-atherosclerotic effects of pitavastatin treatment, we retrospectively examined 59 patients who had a history of stroke and had been prescribed pitavastatin for the treatment of dyslipidemia at the Neurology department of Toho University Ohashi Medical Center Hospital. The patients were classified into two groups according to their homocysteine levels. Carotid artery plaque progression was determined before and after pitavastatin treatment. Plasma levels of high-sensitivity C-reactive protein, platelet molecular markers, and von Willebrand factor were measured. Pitavastatin treatment had beneficial effects on the lipid profiles of these patients and slowed atherosclerosis progression. These effects were observed in both the high and low homocysteine groups. Proactive lipid intervention using pitavastatin may inhibit the progression of atherosclerosis and contribute to secondary prevention of stroke in high-risk patients. We conclude that this statin could inhibit progression at any stage of disease and should therefore be proactively administered to these patient groups, regardless of disease severity.

## Introduction

The prevention of recurrent stroke is extremely important after a transient ischemic attack or initial stroke, and antiplatelet drugs are administered prophylactically in many cases. Dyslipidemia is a risk factor for stroke, and higher low-density lipoprotein cholesterol (LDL-C) levels have been reported to increase the risk of stroke [1]. The American Heart Association/American Stroke Association guidelines [2] recommend proactive LDL-C management by statin administration to prevent stroke recurrence, and this has become the standard approach to the care of these patients. However, there is less evidence for the efficacy of dyslipidemia countermeasures for stroke prevention in Japan, as compared to other countries, and they may be insufficient in this country.

A higher ratio of LDL-C: high-density lipoprotein cholesterol (HDL-C) (L/H ratio) increases platelet activation and elevates the risk of thrombosis. In addition, the independent atherosclerosis-promoting factor, homocysteine, is a marker that reflects the degree of progression of atherosclerosis from its early stages. The L/H ratio and the homocysteine level affect each other and promote progression of atherosclerosis. Conversely, progression of this disease would be expected to be inhibited on reduction of the L/H ratio. However, it is not known if drug efficacy is affected by the degree of disease progression.

We therefore conducted this study with the objective of assessing the anti-atherosclerotic effects of pitavastatin in secondary prevention stroke patients who were divided into a high homocysteine group and a low homocysteine group, as a marker of the degree of atherosclerosis progression.

The study was conducted by measuring inflammatory makers such as high-sensitivity C-reactive protein (hs-CRP), platelet molecular markers (β-thromboglobulin [βTG] and platelet factor 4 [PF4]), and vascular endothelium injury markers such as von Willebrand factor (vWF). βTG is a platelet-specific globulin that accounts for 10% of platelet α granule molecules. PF4 is a chemokine that is expressed specifically in megakaryocytes and contributes to inflammation and wound healing. βTG and PF4 accumulate in platelet α granules and are extremely important indicators of activation. vWF is produced in vascular endothelial cells and megakaryocytes. It facilitates platelet adhesion to damaged vascular subendothelial tissue and thus has an important role in primary hemostasis. Levels of CRP, βTG, PF4, and vWF before and after pitavastatin administration were compared retrospectively.

## Materials and Methods

### Study Population

The subjects of this retrospective survey were 59 patients with a history of stroke who were examined and administered 1–2 mg/day of pitavastatin for treatment of dyslipidemia at the Neurology department of Toho University Ohashi Medical Center Hospital between 2006 and 2011. This study was approved by the Toho University Ohashi Medical Center Institutional Review Board (Confirmation No. 12-35) and all patients gave written informed consent before they were enrolled in the study. The mean administration period was 19 months. Patients were divided into two groups, according to their homocysteine levels. Group H consisted of patients whose homocysteine level was above the median, and Group L consisted of patients whose homocysteine level was below the median.

### Blood Sampling and Biochemical Assays

Blood was carefully drawn by a small number of skilled medical technicians to minimize the impact of this process on the platelet molecular markers. Total cholesterol (TC), HDL-C, triglycerides (TG), L/H ratio, non-HDL-C, homocysteine, βTG, PF4, vWF, and hs-CRP were measured before and after pitavastatin administration. LDL-C was calculated using the Friedewald equation. Non-HDL-C was calculated by subtracting HDL-C from TC. Plasma homocysteine levels were determined using the AxSYM immunoassay (Abbott Laboratories, Abbott Park, Ill.), according to the manufacturer's instructions. βTG and PF4 were measured in the plasma samples by the Asserachrom enzyme-linked immunoassay (ELISA) (β-TG, cat. no. 11875370011; PF4, cat. no. 11875353011, Roche Diagnostics). vWF was measured by ELISA as described previously [3]. Hs-CRP was analyzed on a Modular Analytics P800 using Tina-quant reagents (cat. no. 11972855, Roche Diagnostics). The numbers of patients where βTG, PF4, vWF, homocysteine, and hs-CRP were successfully measured before and after pitavastatin administration were 35, 35, 11, 32, and 43, respectively.

### Carotid ultrasonography

Carotid ultrasonography was performed for each study subject. The mean intima-media thickness (IMT) of the common carotid artery was calculated as the mean value of the maximum IMT and those measured at points 1 cm before and after it. The mean of these values (mean IMT) was determined successfully in 14 patients before and after pitavastatin administration. The plaque score was also determined in 13 subjects. Using the internal/external arterial bifurcation as a standard, the area from the distal side to the proximal side was segmented into four parts at 15-mm intervals. The plaque score was calculated from the sum of the max IMT of 1.1 mm or above on both the carotids.

### Statistical Analysis

Data were analyzed by a one-sample t-test or Wilcoxon signed rank sum test, and analysis of variance (ANOVA) was used for inter-group testing. In all tests, a two-tailed significance level of 5% was used, and *p*<0.05 was therefore considered to indicate a significant difference.

## Results

### Subject Demographics

Information relating to the 59 patients enrolled in this study is presented in Table 1. Antiplatelet drugs were administered to 28 patients and two antiplatelet drugs were used together in four individuals. No vitamin supplements were used by the patients during statin treatment. There were no significant differences between the parameters presented in Table 1 for the H and L homocysteine groups.

**Table 1:** Patient Samples.

			All	Group L	Group H
Sample size			59	16	16
Mean age (range)			66 years (45–89 years)	64.1 years (48–82)	68.7 years (48–89)
Gender	Male		35 (59.3%)	7 (43.8%)	11 (68.8%)
Stroke type	Lacunar stroke		45 (76.3%)	12 (75.0%)	10 (62.5%)
	Non-lacunar stroke		14 (23.7%)	4 (25.0%)	6 (37.5%)
Complications	Hypertension		27 (45.8%)	5 (31.3%)	10 (62.5%)
	Diabetes		6 (10.1%)	0 (0.0%)	2 (12.5%)
	Hyperuricemia		8 (13.6%)	2 (12.5%)	5 (31.3%)
Antiplatelet drugs	Aspirin		13 (22.0%)	5 (31.3%)	3 (18.8%)
	Aspirin, dihydroxyaluminum aminoacetate, magnesium carbonate		7 (11.9%)	1 (6.3%)	3 (18.8%)
	Sarpogrelate hydrochloride		9 (15.3%)	1 (6.3%)	5 (31.3%)
	Cilostazol		2 (3.4%)	0 (0.0%)	1 (6.3%)
	Clopidogrel sulfate		1 (1.7%)	1 (6.3%)	0 (0.0%)

### Changes in Serum Lipids after Pitavastatin Administration

**Figure 1:**
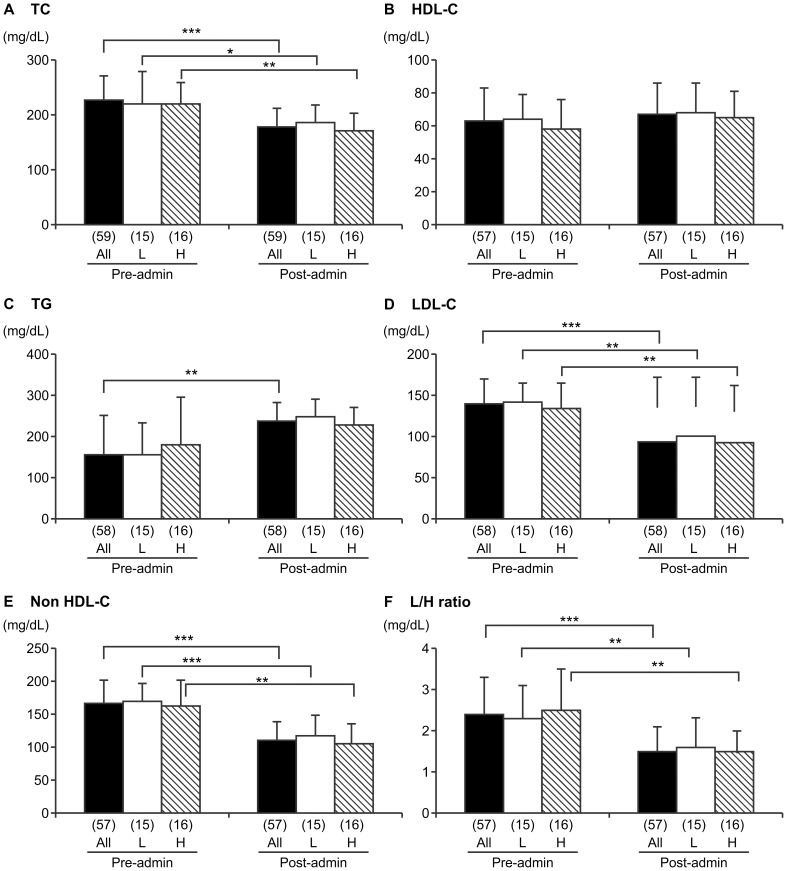
Lipid levels before and after pitavastatin treatment. The mean ± standard deviation is presented. H indicates the group with homocysteine values above the median (8.6 µmol/L), L indicates the group with homocysteine values <8.6 µmol/L. The number of subjects is indicated in parentheses. a) total cholesterol (TC), b) high-density lipoprotein cholesterol (HDL-C), c) triglyceride (TG), d) low-density lipoprotein cholesterol (LDL-C), e) non-HDL-C, and f) the L/H ratio. Pre- and post-pitavastatin values were compared as indicated by t-test or Wilcoxon signed-rank test (TG only). *p<0.05, **p<0.01, ***p<0.001.

Serum lipid levels (TC, LDL-C, HDL-C, TG, non-HDL-C, and L/H ratio) before and after pitavastatin administration are shown in Fig. 1. In all patients, significant improvements in their lipid profiles were seen after pitavastatin administration, with mean TC (± standard deviation, SD) decreasing from 227±44 mg/dL to 178±34 mg/dL (*p*<0.0001), LDL-C from 139±30 mg/dL to 93±28 mg/dL (*p*<0.0001), and TG from 155±95 mg/dL to 125±85 mg/dL (*p* = 0.004). Although not significant, HDL-C tended to increase, rising from 63±20 mg/dL to 67±19 mg/dL (*p* = 0.1747).

The L/H ratio decreased significantly from 2.4±0.9 to 1.5±0.6 (*p*<0.0001) during pitavastatin administration and non-HDL-C also decreased significantly from 167±35 mg/dL to 111±29 mg/dL (*p*<0.0001). No significant difference was observed between the H and L groups of subjects divided on the basis of the median homocysteine level, which was 8.6 µmol/L. Significant improvements in the lipid profiles of both subgroups were seen following pitavastatin administration, except for TG and HDL-C.

### Effects of Pitavastatin on Markers in the Homocysteine Subgroups

**Figure 2:**
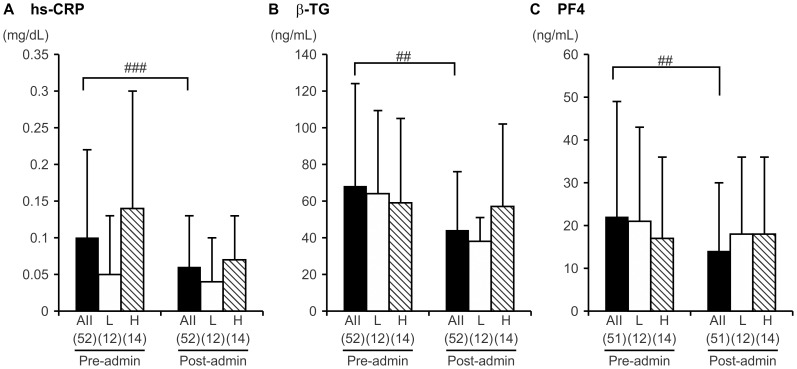
Inflammatory and platelet activity markers before and after pitavastatin treatment. Mean ± standard deviation is shown. H indicates the group with homocysteine values above the median (8.6 µmol/L), L indicates the group with homocysteine values <8.6 µmol/L. The number of subjects is indicated in parentheses. a) high-sensitivity C-reactive protein (hs-CRP), b) β-thromboglobulin (βTG), and c) platelet factor 4 (PF4). Pre- and post-pitavastatin values were compared by Wilcoxon signed-rank test. ##*p*<0.01, ###*p*<0.001.

**Figure 3:**
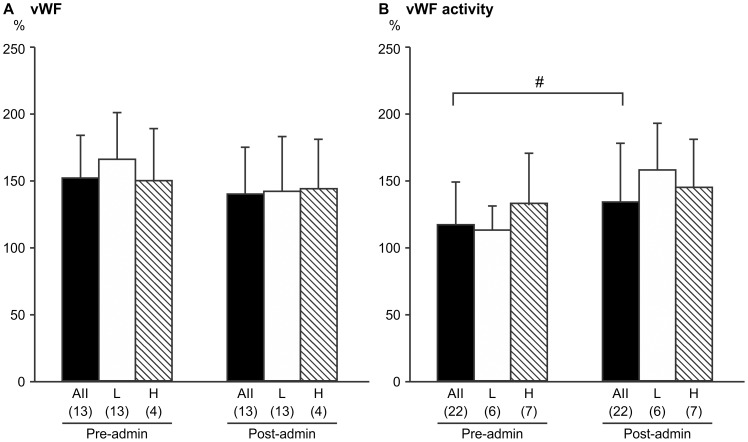
Levels and activity of von Willebrand factor (vWF) before and after pitavastatin treatment. Mean ± standard deviation is shown for a) vWF levels and b) vWF activity. H indicates the group with homocysteine values above the median (8.6 µmol/L), L indicates the group with homocysteine values <8.6 µmol/L. The number of subjects is indicated in parentheses. Pre- and post-pitavastatin values were compared using the Wilcoxon signed-rank test. #*p*<0.05

The levels of hs-CRP, βTG, PF4, and vWF before and after pitavastatin administration are shown in Fig. 2 and Fig. 3. Significant improvements in the levels of the inflammatory marker, hs-CRP, were seen after administration, as this decreased from 0.10±0.12 mg/dL to 0.06±0.07 mg/dL (*p*<0.0001, Fig. 2a). Both platelet molecular markers exhibited significant improvement, with βTG decreasing from 68±56 ng/dL to 44±32 ng/dL (*p* = 0.0049, Fig. 2b), and PF4 decreasing from 22±27 ng/dL to 14±16 ng/dL (*p* = 0.012, Fig. 2c). When subjects were divided into H and L groups based on their homocysteine level, no significant differences were observed in hs-CRP, βTG or PF4 before and after pitavastatin treatment, although a trend for βTG to decrease was seen in the L group.

There was no significant pitavastatin-related change in the level of vWF, which decreased from 152±32% to 140±35% (*p* = 0.471, Fig. 3a), although a significant increase in the activity of this endothelial marker was observed, from 117±32% to 134±44% (*p* = 0.01, Fig. 3b). No significant differences in vWF were observed between the H and L homocysteine groups.

### Plaque Scores in the Homocysteine Subgroups

**Figure 4:**
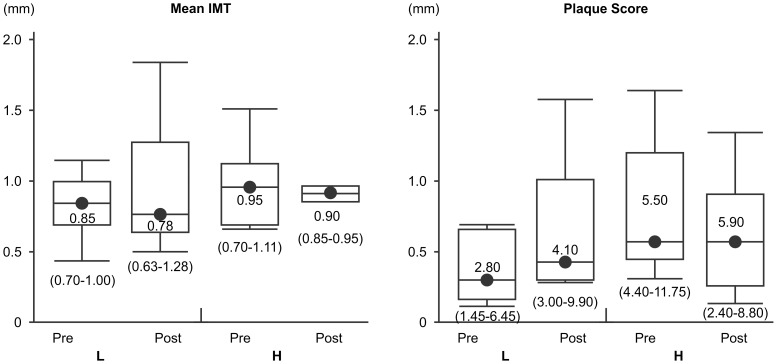
Intima-media thickness (IMT) and plaque scores before and after pitavastatin treatment. H indicates the group with homocysteine values above the median (8.6 µmol/L), and L indicates the group with homocysteine values <8.6 µmol/L. The median values and the range (first quartile to third quartile) are presented. Paired t-tests were performed but no significant differences were observed.

The mean IMT and plaque scores before and after administration in the H and L homocysteine groups are shown in Fig. 4. Mean IMT and plaque scores before pitavastatin administration tended to be higher in the H group, although these differences were not statistically significant (*p* = 0.7, *p* = 0.11, respectively). Mean IMT exhibited a decreasing trend in the H group, falling from a median value of 0.95 (0.70–1.11: first quartile–third quartile) mm before administration, to 0.90 (0.85–0.95) mm after administration. A similar decline was observed in the L group, from a median value of 0.85 (0.70–1.00) mm to 0.78 (0.63–1.28) mm, but neither of these differences were statistically significant. In the H group, plaque score shifted from a median value of 5.5 (4.40–11.75) before administration to 5.9 (2.40–8.80) after administration, and in the L group this was 2.8 (1.45–6.45) before, and 4.1 (3.00–9.90) after, pitavastatin administration.

### Safety

No clinically problematic changes in laboratory test values or symptoms were seen throughout the pitavastatin administration period, and no adverse events were reported.

## Discussion

In this study, we examined whether pitavastatin produced different effects in patients with advanced atherosclerosis and those without, using homocysteine as an indicator. It has been reported that individuals with a high blood homocysteine concentration have an elevated risk for atherosclerotic disease and for death due to cerebro-cardiovascular disease [4]. Furthermore, in the Japanese population, elevated homocysteine level associated with an increased mean carotid artery IMT [5], and an association has also been reported with the risk for ischemic and lacunar stroke [6]. Homocysteine acts on vascular endothelial cells, amplifying the expression of 3-hydroxyl-3-methylglutaryl coenzyme A reductase, stimulating cholesterol production, and inhibiting nitric oxide production [7]. However, it has not yet been demonstrated that supplementation with folic acid and vitamins B6 and B12, which reduce homocysteine levels, can reduce the risk of stroke [8]. Previous studies reported that statins did not affect the homocysteine level [9] but partially inhibited homocysteine-mediated exacerbation of atherosclerosis [10], and showed that proactive lipid intervention using high doses of statins is critical for stroke prevention: however, only a few, studies have analyzed the efficacy of high dose statins for stroke prevention. The present study found no significant differences in the effects of pitavastatin administration on blood lipids, hs-CRP, platelet molecular markers, or vWF in the H and L homocysteine groups. We also considered the relationship between platelet molecular markers and homocysteine level. Since βTG tended to decrease after pitavastatin administration in the L group, pitavastatin may be more effective when started at the early stages of the disease, when relatively mild atherosclerotic changes are present. Pitavastatin was found to inhibit plaque progression, with no clear differences in the effect of this statin on the IMT and plaque scores in the H and L homocysteine groups. Antioxidant capacity has been reported to be weak in patients with high blood homocysteine concentrations [11], while lipid intervention using statins has direct anti-inflammatory [12] and antioxidant effects [13]. In the present study, our findings indicated that pitavastatin-related improvements in lipid profiles inhibited progression of atherosclerosis, even in patients at high risk of plaque progression.

In contrast to the situation with cardiovascular disease, there is little evidence regarding the influence of lipid levels in primary and secondary stroke prevention, and LDL-C is not managed as stringently as it is in cardiovascular disease. Furthermore, in stroke patients with the complication of hypertension, blood pressure management is considered to be more important than lipid-lowering therapy and it is difficult to actually prevent stroke by lipid management alone. While the subjects of this study were extremely high-risk post-stroke patients, the fact that we observed an inhibition of carotid artery plaque progression following pitavastatin administration clearly indicated the importance of lipid management using statins in these patients.

Because the sample size was limited in this study, we could not investigate whether inhibition of plaque progression led to stroke prevention. We were also unable to ascertain the effect of pitavastatin administration on homocysteine levels. We hope to clarify these points by conducting a long-term large-scale study in the future.

## Conclusions

Proactive lipid intervention using pitavastatin may inhibit progression of atherosclerosis and contribute to secondary prevention of stroke in high-risk patients. Although this intervention may be particularly useful at the early stages of the disease when homocysteine levels are low, we conclude that it could inhibit progression at any stage and should therefore be proactively administered to these patient groups, regardless of their disease severity.
